# *SLC11A1* polymorphisms and host susceptibility to cutaneous leishmaniasis in Pakistan

**DOI:** 10.1186/s13071-016-1934-2

**Published:** 2017-01-07

**Authors:** Mariam Sophie, Abdul Hameed, Akhtar Muneer, Azam J. Samdani, Saima Saleem, Abid Azhar

**Affiliations:** 1 Karachi Institute of Biotechnology & Genetic Engineering (KIBGE), University of Karachi, Karachi, 75270 Pakistan; 2 Institute of Biomedical and Genetic Engineering (IBGE), 24-Mauve Area, G- 9/1, Islamabad, Pakistan; 3 Kuwait Teaching Hospital, Abdara Chowk, University Road, Peshawar, Pakistan; 4 National Medical Centre, A-5/A, National Highway, Phase 1, Defence Housing Authority, Karachi, Pakistan

**Keywords:** Cutaneous leishmaniasis, *SLC11A1*, Single nucleotide polymorphism, Genetic susceptibility, Pakistan

## Abstract

**Background:**

The vector-borne cutaneous leishmaniasis (CL) is endemic in several regions of Pakistan mainly affecting poor populations. Host genetic factors, particularly *SLC11A1* (solute carrier transmembrane protein) within macrophages, play a crucial role in disease pathology and susceptibility. Association of *SLC11A1* with cutaneous leishmaniasis, a neglected tropical disease, is not well established. Inconsistencies have been observed within different populations worldwide with respect to genetic susceptibility. This study was designed to investigate genetic variation(s) in *SLC11A1* and to assess possible association with cutaneous leishmaniasis in Pakistan.

**Results:**

Eight polymorphisms (rs2276631, rs3731864, rs2290708, rs2695342, rs201565523, rs17215556, rs17235409, rs17235416) were genotyped across *SLC11A1* in 274 patients and 119 healthy controls. Six polymorphisms were studied by polymerase chain reaction-restriction fragment length polymorphism (PCR-RFLP) and sequencing. Two single nucleotide polymorphisms were analyzed with newly designed semi-nested PCR assays. Case-control analysis showed no association between selected polymorphisms in *SLC11A1* and cutaneous leishmaniasis. No significant difference was observed in the distribution of alleles between leishmaniasis patients and healthy individuals. Strong pairwise linkage disequilibrium was observed between rs2276631 and rs2290708 (*r* ^2^ = 64); and rs17235409 and rs17235416 (*r* ^2^ = 78).

**Conclusions:**

This study shows that genetic variations in the candidate gene *SLC11A1* do not affect susceptibility to cutaneous leishmaniasis in the sample population from Pakistan.

**Electronic supplementary material:**

The online version of this article (doi:10.1186/s13071-016-1934-2) contains supplementary material, which is available to authorized users.

## Background

Leishmaniasis is a group of diseases with diverse clinical outcomes that range from self-healing ulcerative lesions on the skin (cutaneous leishmaniasis) to fatal visceral infection. This vector-borne tropical disease is caused by the digenetic parasitic protozoa, *Leishmania*, which exists as either flagellated promastigotes in *Phlebotomus* sand fly vectors or non-flagellated amastigotes in phagocytic cells. Disease manifestation depends upon the encounter between the invading protozoa and host organism leading to either susceptibility or resistance to the infection [1, 2]. There are over 20 different *Leishmania* species and more than 90 sand fly species responsible for leishmaniasis and parasite transmission, respectively. According to World Health Organization statistics, leishmaniasis is endemic in 98 countries, endangering 350 million people. It has an incidence of 1.3 million cases with an estimated mortality rate of 20,000 to 30,000 per year [3, 4].

In Pakistan, the visceral form of leishmaniasis is mainly restricted to the Azad Jammu Kashmir and Abbottabad regions in the north [5, 6]. The major burden lies in the form of cutaneous leishmaniasis which is reported from all parts of the country, particularly Balochistan and Khyber Pakhtunkhwa Provinces [7] with a significant proportion found in children aged 14 years or less [8]. The influx of refugees from Afghanistan along the western border of Pakistan is considered as one of the contributing factors responsible for the growing number of cases in this region [9].

Host genetics, in addition to the infecting *Leishmania* species, parasite load and environmental factors, play a crucial role in determining the type and severity of the disease [10]. Genome wide association studies have identified solute carrier family 11 member a1 (*SLC11A1*) gene as a strong candidate which assists in intracellular pathogen control [11]. *SLC11A1* gene spans 12 kb in length comprising 15 exons. These encode a 550 amino acid protein with 10–12 predicted transmembrane domains [12]. It is localized to the phagosome membrane and is involved in the transport of divalent cations [13]. During an intracellular infection, SLC11A1 transports essential elements (Mn^2+^, Fe^2+^, Co^2+^) vital for the survival of the parasite, from the phagolysosome into the cytosol and hence starving and restricting their growth [14]. Nucleotide analysis of *SLC11A1* gene in inbred mice strains revealed a single non-synonymous amino acid substitution of glycine to aspartic acid (Gly169Asp). Mice with this mutation were unable to produce a functional protein which made them susceptible to intracellular parasites [15]. This non-conservative mutation has not yet been identified in the human homologue SLC11A1.

Restriction and subsequent resolution of intracellular parasites following phagocytosis by macrophages has made SLC11A1 a strong candidate for predisposition to different infectious diseases like tuberculosis and leprosy [16, 17]. Genetic analysis and sequencing have identified multiple genetic polymorphisms within the human homologue SLC11A1 [18]. However, these genetic variations when studied with respect to susceptibility to intracellular *Leishmania* protozoa reveal an inconsistent pattern across different regions of the world [18–25]. Therefore, the current study was designed to determine and analyze the genetic variation(s) in *SLC11A1* gene and investigate if these polymorphism(s) are associated with cutaneous leishmaniasis in Pakistan.

## Methods

### Sample collection

Samples for this study were collected with informed consent over the course of three years (2010 to 2012). Subjects included 274 clinically diagnosed leishmaniasis patients presenting to local hospitals of Karachi (Jinnah Postgraduate Medical Centre and Sindh Institute of Skin Diseases) and Peshawar (Kuwait Teaching Hospital). Diagnosis was based on the direct microscopic visualization of stained *Leishmania* amastigotes from lesion exudates. Controls comprised a total of 119 healthy contacts exposed to the same environment as the patients. Patients from both genders and all ages were included in the study.

### DNA isolation

Blood samples (5 ml) were collected using acid citrate dextrose (ACD) vacutainers to prevent coagulation. Genomic DNA was extracted from peripheral blood leukocytes by standard phenol-chloroform method [26]. Concentration of isolated DNA was determined by spectrophotometric analysis (Analytik Jena, Jena, Germany). Genomic DNA concentrations of 50 ng/μl were prepared of all samples for genotyping analysis and stored at -20 °C till further processing.

### Primer design and polymerase chain reaction

Whole gene sequence of *SLC11A1* was retrieved from Ensembl database. Intron specific primers were designed which allowed the amplification of whole or multiple exons, depending on their size using the online software Primer3 [27]. Polymerase chain reaction (PCR) was optimized for each primer set including annealing temperature and PCR master mix concentrations. A typical PCR reaction consisted of 30 μl reaction mixture containing 1× PCR buffer, 1.5–3 mM MgCl_2_, 200 μM deoxynucleotide triphosphates (dNTPs) each, 0.4 μM of each forward and reverse primer and 1–3 units of Taq polymerase (Thermo Scientific, Waltham, USA). PCR cycle conditions included an initial denaturation step at 94 °C for 4 min followed by 35 cycles consisting of: denaturation at 94 °C for 30 s, annealing at 55–58 °C (specific for each primer set) for 35 s and elongation at 72 °C for 30 s, followed by a final elongation step at 72 °C for 7 min (ThermoHybaid, Needham, USA). Five microliters of PCR product was loaded onto 2% agarose gel stained with ethidium bromide (0.5 μg/ml) for electrophoresis. The PCR products were visualized under ultraviolet (UV) illumination using Gel-Documentation System (Bio-Rad, Hercules, USA).

### Sequence analyses

Preliminary sequence analyses of at least 25 PCR samples of each amplified exon was carried out. Prior to sequencing, PCR products were purified by *AccuPrep* PCR Purification Kit (Bioneer, Daejeon, Korea) using a binding column tube. The purified products were assessed on 2% agarose gel to confirm a successful purification reaction. These were then sequenced using ABI 3730XL DNA Analyzer (Applied Biosystems, Waltham, USA). Sequenced products were later aligned and analyzed using the Molecular Evolutionary Genetics Analysis (MEGA) software [28].

### Genotyping

Sequenced products were scanned for DNA sequence variations including substitutions, insertions or deletions. Eight nucleotide variations were identified. These included two silent substitutions; one in exon 3 (274C/T) and the other in exon 9 (825A/G); a substitution in intron 5 (577-18G/A) and intron 7 (639 + 22C/T); 3 missense mutations (A318V in exon 9, V443A in exon 13, D543N in exon 15) and a four base pair TGTG deletion (1729 + 55del4) in the 3′ untranslated region (3′UTR). Based on these polymorphisms, restriction fragment length polymorphism (RFLP) experiments were designed.

### Restriction fragment length polymorphism (RFLP)

RFLP was performed for six of the above mentioned single nucleotide polymorphisms (SNPs) for verification of these SNPs in the remaining sample population (Additional file 1: Table S1). These included rs2276631 (274C/T) in exon 3, rs3731864 (577-18G/A) in intron 5, rs2695342 (825A/G) in exon 9, rs201565523 (A318V) in exon 9, rs17235409 (D543N) in exon 15 and rs17235416 (1729 + 55del4) in 3′UTR. Restriction enzymes for each SNP were selected using NEBcutter V2.0 [29]. A total volume of 30 μl reaction containing 10 μl of PCR product, 2 μl of 10× buffer and 1 μl restriction enzyme was prepared. PCR products were digested overnight at 37 °C in a water bath according to manufacturer’s instructions (Fermentas, Waltham, USA). The obtained restriction digests were resolved on 2.5% agarose gel by electrophoresis. The ethidium bromide stained gels were visualized under UV illumination.

### Semi-nested PCR

Direct genotyping of the two SNPs, rs2290708 (639 + 22C/T) and rs17215556 (V443A) in intron 7 and exon 13, respectively, was performed by designing two semi-nested PCR assays, one for each SNP. Allele specific primers were designed using PRIMER1 [30].

Semi-nested PCR for the SNP 639 + 22C/T in intron 7 was performed with a total volume of 15 μl reaction. The reaction contained 200 ng of genomic DNA, 0.4 μM of each forward outer primer (5′-CTG CGG AAG CTG GAA GCT TTT TTT GGA C-3′) and T-allele specific reverse inner primer (5′-GAG GAA TGA TCT TGG GAG GTC CAC AGT TGA-3′), 0.2 μM of reverse outer primer (5′-CAC ATA CTG CAG GGA GGA GCC TGG TCA G-3′), 200 μM dNTPs each, 1.2 mM MgCl_2_, 1× PCR buffer (400 mM KCl and 100 mM Tris-HCl, pH 9.0) and 1 unit of *Taq* DNA polymerase (Bioneer). Amplification was performed using Multi Block System 0.2S (ThermoHybaid) with the following conditions: initial denaturation at 94 °C for 4 min, followed by 35 cycles comprising denaturation at 94 °C for 30 s, annealing at 56 °C for 35 s and extension at 72 °C for 30 s. The last cycle was followed by a final extension at 72 °C for 7 min.

Similarly, semi-nested PCR for the SNP V443A in exon 13 was performed in a total reaction of 15 μl containing 200 ng genomic DNA, 1× PCR buffer, 1.5 units *Taq* polymerase (Thermo Scientific), 1.2 mM MgCl_2_, 200 μM dNTPs each, 0.2 μM forward outer primer (5′-CAG TTG AGC TCA CAC CCA CAC A-3′), 0.1 μM reverse outer primer (5′-ACC TCT CTC AGC CTC TGT GGC T-3′) and 0.2 μM C-allele specific reverse inner primer (5′-AAC GTG AGG ATG GGC ATC G-3′).

A touch down semi-nested PCR approach was adopted for the SNP V443A. PCR conditions included an initial denaturation step at 94 °C for 4 min. The first 15 cycles out of 35 cycles were run at the following conditions: 94 °C for 30 s for denaturation, 58 °C for 35 s for annealing and 72 °C for 30 s for elongation. The cycling conditions for the remaining 20 cycles included: denaturation at 94 °C for 30 s, annealing at 55 °C for 35 s and elongation at 72 °C for 30 s. The PCR reaction ended with a final elongation at 72 °C for 7 min.

The PCR products from both semi-nested PCR assays were run on ethidium bromide stained 2.5% agarose gel and bands were observed by means of a gel documentation system (Bio-Rad).

### Statistical analyses

Genotypic and minor allele frequencies (MAF) were calculated for each SNP among cases and controls. Statistical tests including Chi-square test and odds ratio (OR) with 95% confidence interval were performed for association analysis of *SLC11A1* gene with cutaneous leishmaniasis using PLINK v1.07 software under the null hypothesis of “no association” [31]. Pairwise linkage disequilibrium plots of the SNPs were created using Haploview 4.2 [32]. Power calculations for the genetic study was undertaken following the guidelines elaborated by Sham and Purcell [33]. *P-*values less than 0.05 were considered statistically significant.

## Results

### Sequence analyses

Nucleotide sequences of purified PCR samples were analyzed by means of multiple sequence alignment with MEGA software. These sequences were compared with the retrieved *SLC11A1* gene sequence from Ensembl database. Alignment revealed eight nucleotide polymorphisms spread over exon 3, intron 5, intron 7, exon 9, exon 13, exon 15 and 3′UTR regions, respectively, some of which had been described previously [18, 20]. The newly designed primers which covered whole exons did not detect any new functional polymorphisms in the following coding regions: exon 4, 5, 6, 7, 10, 11 and 12. The non-conservative glycine to aspartic acid substitution formerly reported in mice was also not detected. The total genetic variants identified in *SLC11A1* are described in Table 1.

**Table 1:** *SLC11A1* polymorphisms

Genetic variation	Location	SNP identity	Nucleotide change^a^	Amino acid change	Observed alleles^b^
274C/T	Exon 3	rs2276631	TT**C** → TT**T**	Phe → Phe	C/T
577–18G/A	Intron 5	rs3731864	G → A	–	G
639 + 22C/T	Intron 7	rs2290708	C → T	–	C/T
825A/G	Exon 9	rs2695342	GC**A** → GC**G**	Ala → Ala	G
A318V	Exon 9	rs201565523	G**C**G → G**T**G	Ala → Val	C
V443A	Exon 13	rs17215556	G**T**G → G**C**G	Val → Ala	T/C
D543N	Exon 15	rs17235409	**G**AC → **A**AC	Asp → Asn	G/A
1729 + 55del4	3′UTR	rs17235416	TGTG deletion	–	[+/−]^c^

^a^Characters in bold indicate the nucleotide change reported. SNP identity retrieved from dbSNP Short Genetic Variations

^b^Alleles identified in the sample population

^c^ + = insertion of TGTG; − = deletion of TGTG

### *SLC11A1* genotyping

The SNPs identified through initial sequence analysis either created or destroyed a restriction site. Identification of polymorphisms in the remaining number of samples in the given population was thus carried out by restriction fragment length polymorphism analysis for the SNPs rs2276631 (274C/T) in exon 3, rs3731864 (577-18G/A) in intron 5, rs2695342 (825A/G) and rs201565523 (A318V) in exon 9, rs17235409 (D543N) in exon 15 and rs17235416 (1729 + 55del4) in 3′ UTR as described by Liu et al. [18]. The alleles of each polymorphism presented a distinct pattern of restriction digests with agarose gel electrophoresis after treatment with restriction enzymes.

The two newly-designed semi-nested PCR assays for the direct identification of the SNPs in intron 7 and exon 13 produced two bands with agarose gel electrophoresis; a larger fragment produced by outer primers and one smaller fragment corresponding to the specific allele.

Semi-nested PCR for rs2290708 (639 + 22C/T) gave a band at 239 bp with the outer primers for all samples. A second band of 117 bp was observed in only those samples which were T-allele positive. Similar results were observed for rs17215556 (V443A) polymorphism specific semi-nested PCR assay. A larger band at 306 bp with the outer primers was observed for each and every sample. While a second smaller band at 143 bp specific for the rarer C-allele was observed only in those samples that carried the C-allele.

Table 2 lists all the possible genotypes for each polymorphism and summarizes the genotypic and MAF distribution among cases and controls for all genetic variations. In addition, MAF reported from other populations are also listed for comparison. All SNPs were in Hardy-Weinberg equilibrium for controls. The polymorphisms rs3731864 (577-18G/A), rs2695342 (825A/G) and rs201565523 (A318V) were monomorphic exhibiting only GG, GG and CC genotype, respectively. Two genotypes, CC and CT, were observed for the SNP rs2276631 (274C/T) with the genotypic frequencies exhibiting no significant difference between cases and controls. Variations in exon 13 (rs17215556) and exon 15 (rs17235409) displayed similar results with only two genotypes present in the sample population. TT and CT genotypes were identified for rs17215556 while genotypes GG and GA for rs17235409, respectively. Genotyping for the SNP rs2290708 (639 + 22C/T) in intron 7 detected all three genotypes (CC, CT, and TT) in both cases and controls. Homozygous 4 base pair insertion (TGTG+/TGTG+) at 3′ UTR (rs17235416) was more common among cases and controls as compared to homozygous deletion (TGTG-/TGTG-). Additionally, the heterozygous genotype (TGTG+/TGTG-) was only observed in cases and none in controls.

**Table 2:** *SLC11A1* polymorphisms and comparison of MAF with other populations from HapMap and dbSNP

Polymorphism	Region	Genotype	Cases (%)	Controls (%)	A1^a^	A2^b^	MAF^c^	Caucasian	Asian	African
274C/T	Exon 3	CC	206 (75.2)	86 (72.3)	T	C	0.1272	0.216	0.112	0.158
		CT	68 (24.8)	33 (27.7)						
		TT	0	0						
577–18G/A	Intron 5	GG	274 (100)	119 (100)	–	G	–	0.053	0.062	0.139
		GA	0	0						
		AA	0	0						
639 + 22C/T	Intron 7	CC	208 (75.9)	88 (74.0)	T	C	0.1361	0.212	0.698	0.1195
		CT	57 (20.8)	30 (25.2)						
		TT	9 (3.3)	1 (0.8)						
825A/G	Exon 9	AA	0	0	–	G	–	0.001	0.0	0.056
		AG	0	0						
		GG	274 (100)	119 (100)						
A318V	Exon 9	CC	274 (100)	119 (100)	–	C	–	0.0	0.0	0.0
		CT	0	0						
		TT	0	0						
V443A	Exon 13	TT	262 (95.62)	110 (92.44)	C	T	0.027	0.005	0.0167	0.0015
		CT	12 (4.38)	9 (7.56)						
		CC	0	0						
D543N	Exon 15	GG	252 (91.97)	115 (96.64)	A	G	0.033	0.009	0.088	0.058
		GA	22 (8.03)	4 (3.36)						
		AA	0	0						
1729 + 55del4	3′UTR	TGTG (+/+)	250 (91.2)	113 (94.96)	(−)	(+)	0.042	0.01^e^	0.18^e^	0.31^f^
		TGTG (+/−)^d^	1 (0.36)	0						
		TGTG (−/−)	23 (8.39)	6 (5.04)						

^a^Minor allele

^b^Major allele

^c^Minor allele frequency

^d^ + = insertion of TGTG; − = deletion of TGTG

^e^Data from [19]

^f^Data from [21]

### Association of *SLC11A1* and CL

This study included 274 *Leishmania* infected cases and 119 healthy controls exposed to the same environment. Eight SNPs were genotyped, out of which 3 were monomorphic. The remaining five SNPs were tested for association with cutaneous leishmaniasis using PLINK, a genomic association testing toolset. Table 3 shows the frequency distribution of the minor alleles among cases and controls. Single locus association test confirmed that the null hypothesis of “no association” with cutaneous leishmaniasis holds true for all markers tested (Table 3).

**Table 3:** Single locus association test between *SLC11A1* polymorphisms and CL

Polymorphism	A1^a^	F_A^b^	F_U^c^	A2^d^	*P*	OR^e^	95% CI^f^
274C/T	T	0.1223	0.1387	C	0.5263	0.8653	0.553–1.354
639 + 22C/T	T	0.1369	0.1345	C	0.9279	1.021	0.6541–1.593
V443A	C	0.0219	0.03782	T	0.2035	0.5697	0.2368–1.371
D543N	A	0.04015	0.01681	G	0.1271	2.447	0.8339–7.179
1729 + 55del4	–^g^	0.04562	0.03361	+^h^	0.5624	1.374	0.6107–3.093

^a^Minor allele code

^b^Frequency of minor allele in cases

^c^Frequency of minor allele in controls

^d^Major allele code

^e^Odds ratio

^f^95% confidence interval

^g^Deletion of TGTG

^h^Insertion of TGTG

### Linkage disequilibrium

**Fig. 1:**
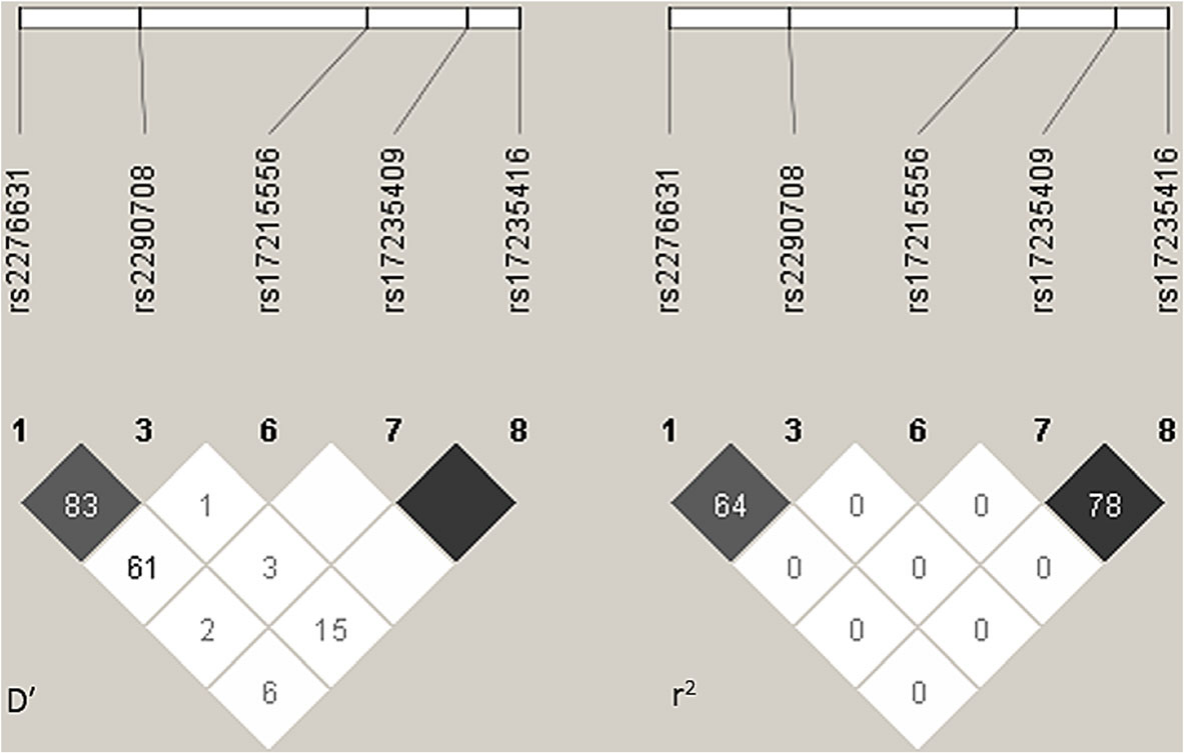
Linkage disequilibrium (LD) plot for the genotyped variations in *SLC11A1* created by Haploview 4.2. For D′ LD plot, confidence bounds color scheme was used: *white*, strong evidence of recombination; *light grey*, uninformative; *dark grey*, strong evidence of LD. *r* ^*2*^ values are represented as *black* for *r* ^2^ = 1; *white* for *r* ^2^ = 0; shades of *grey* indicating values within the range 0 < *r* ^2^ < 1. Numbers within the squares correspond to D′ and *r* ^2^ scores for pairwise LD

The five polymorphic SNPs (274C/T, 639 + 22C/T, V443A, D543N and 1729 + 55del4) were further analyzed for the non-random association of their alleles through the pairwise linkage disequilibrium plot that was generated by Haploview 4.2 and is shown in Fig. 1. The plot shows pairwise D′ and *r* ^2^ scores of linkage disequilibrium (LD) between each SNP of *SLC11A1* with respect to cutaneous leishmaniasis. LD scores between rs2276631 (274C/T) and rs2290708 (639 + 22C/T) was *r* ^2^ = 64. The missense mutation rs17235409 in exon 15 (D543N) and the insertion/deletion polymorphism rs17235416 at 3′UTR (1729 + 55del4) appear to be in strong linkage disequilibrium (*r* ^2^ = 78) whereas the results for the other SNP pairs were either not conclusive or strongly indicated recombination.

## Discussion

Genetic factors play a crucial role in host susceptibility or resistance to infectious diseases. Variations within the *SLC11A1* gene were assessed in this study with respect to cutaneous leishmaniasis in Pakistan which has not been reported before from this region. A total of eight polymorphisms present in *SLC11A1* gene were analyzed and their possible role in disease association and linkage was tested.

Six of these genetic variations (rs2276631, rs2290708, rs3731864, rs201565523, rs17235409 and rs17235416) have been analyzed before in various populations with regard to leishmaniasis. In addition to these six SNPs, two polymorphisms, rs2695342 (825A/G) in exon 9 and rs17215556 (V443A) in exon 13, not investigated previously for their role in cutaneous leishmaniasis, were also included. The SNP rs2695342 (825A/G) in exon 9 alters the codon GCA to GCG. The resultant mutation is synonymous with no change in the amino acid alanine. Only the GG genotype was observed in all samples (cases and controls) unanimously while the genotypes GA and AA were absent from the population. This SNP (rs2695342) lies in the cytoplasmic loop region between transmembrane 6 and transmembrane 7 [34]. Similarly, other monomorphic polymorphisms identified were in intron 5 (577-18G/A) and in exon 9 (A318V), with the latter SNP expected to lie in the extracellular loop region between transmembrane 7 and 8 [34]. The presence of a single genotype in the sample population may suggest homogeneity of the alleles due to the common geographical ancestry within the Pakistani population. The genetic substitution GTG to GCG in exon 13 (rs17215556) is the second SNP whose role has not yet been studied in *Leishmania* patients. This polymorphism causes a modification in codon 443 which results in a change in amino acid valine to alanine in the helical structure of transmembrane 10 [34]. Since this conservative change results in the substitution of one hydrophobic amino acid with another, it is not expected to influence the protein structure. Literature survey shows that association of this non-synonymous mutation with pulmonary *Mycobacterium avium* complex revealed no significant results [35].

Genotyping the SNPs rs2290708 (639 + C/T) in intron 7 and rs17215556 (V443A) in exon 13 was approached by designing a semi-nested PCR. Allele identification through semi-nested PCR for rs17215556 was slightly modified by adding a touch-down step. This introduced two annealing temperatures that improved allele specific amplification. The semi-nested PCR assay made one step genotyping possible therefore making it an efficient technique. All three genotypes (CC, CT, TT) were observed for rs2290708 (639 + C/T) in intron 7 with the rarer TT genotype having a higher frequency in cases as compared to controls. Rarer CC genotype for rs17215556 (V443A) in exon 13, was absent from the population. Other polymorphisms including 274C/T, D543N and 1729 + 55del4 also displayed more than one genotype in the sample population.

Minor allele frequencies were similar to other populations including Caucasians, Africans and Asians except for rs17235416 (1729 + 55del4) where the frequency for TGTG deletion polymorphism at 3′ untranslated region was much higher in African population (0.31) when compared to the population from Pakistan.

Case-control analysis of these two SNPs (rs2695342 and rs17215556) in addition to the 6 genetic variations previously studied in other populations, revealed no evidence of association between these 8 loci of *SLC11A1* and cutaneous leishmaniasis patients from Pakistan. No significant difference in distribution of alleles between cases and controls was observed. The polymorphisms studied do not suggest a direct impact on structural or functional integrity of the transmembrane protein.

Pairwise linkage disequilibrium results showed strong LD between the non-synonymous substitution rs2276631 and SNP rs2290708 in intron 7. The missense mutation D543N in exon 15 and the 4 bp deletion of TGTG nucleotides (1729 + 55del4) in 3′UTR regions towards the 3′ end of the gene also appeared to be in strong linkage disequilibrium indicating non-random segregation at these loci.

To date, only a few papers have addressed the question of susceptibility to leishmaniasis with respect to the gene *SLC11A1*. This lack of attention and funding for research may possibly be because the disease burden is restricted to poverty-stricken and under developed areas. Studies on the association of *SLC11A1* with leishmaniasis exhibit contradictory and inconsistent results with different patterns observed across the world. A study conducted in Sudan reports strong linkage at the *SLC11A1* promoter region but no association between the gene and disease [19]. Similarly, another study in Sudan revealed association of visceral leishmaniasis (VL) with 5′ polymorphisms including (GT)_n_ at the promoter, 274C/T in exon 3 and 469 + 14G/C in intron 4 whereas 3′ polymorphisms at exon 15 and 3′UTR loci did not appear to have any association [20]. However, in a Moroccan population, only exon 15 and exon 8 polymorphic markers influence susceptibility to VL while 5′ end polymorphisms played no role in disease predisposition [24].

This pattern of fluctuation continues across South America where Brazilian patients carrying TGTG insertion allele at 3′UTR showed association with cutaneous leishmaniasis [21]. When CL and VL were both studied in Mexico, *SLC11A1* polymorphisms were associated with only VL and none with CL [25].

Interestingly, the results from Asian nations including Sri Lanka and India were more consistent. The polymorphic markers of *SLC11A1* when analyzed in Sri Lanka and India, failed to show any association with either CL or VL, respectively [22, 23] as observed in this study of Pakistani CL patients. This overlapping pattern of no association among the south Asian countries may be attributed to similar genetic makeup of their populations with respect to the closeness in geography and large scale migration of people particularly between India and Pakistan at the time of independence.

However, other factors should be taken into account. The infecting *Leishmania* species are not the same in these three countries. In Pakistan, CL is highly endemic with *Leishmania tropica* responsible for infection in the northern and *Leishmania major* in the southern areas of the country, respectively. Whereas in Sri Lanka, CL is caused by *Leishmania donovani*. This species is the causative agent of VL in India which is the predominating form of the disease in the country.

A recent report on multilocus microsatellite typing of *Leishmania* isolates obtained from cutaneous leishmaniasis samples from Pakistan identified *L. major* populations that were genetically different from those found in Africa, Central Asia, Iran and Middle East. They propose that this may be attributed to the different vector and animal host species found in this region [36]. Therefore, in addition to host genetic factors, differences in the clinical forms of leishmaniasis and invading *Leishmania* species are also important elements that may affect the variability in disease susceptibility.

There is a need for more *SLC11A1* and cutaneous leishmaniasis association studies to help understand the cellular mechanism better. It also raises questions whether other regulatory genes of the immune system have a role to play in relation to *SLC11A1* and whether it is the combination of multiple genes that is the underlying factor for leishmaniasis susceptibility. The focus on cutaneous leishmaniasis is critical, not only because it is one of the neglected tropical diseases but also has an alarming rate of infecting one person every 20 s in endemic areas [37]. The persisting disfiguring scars left after treatment of the ulcers are associated with social stigma especially among females [38]. Co-infection of leishmaniasis with human immunodeficiency virus (HIV) is another major problem that has emerged. Cases of co-infection from over 35 countries have been reported which add to the burden of the disease [39].

## Conclusion

This preliminary study was designed to investigate the role of *SLC11A1* gene in a Pakistani population infected with cutaneous leishmaniasis which has not been reported before from this region. It was demonstrated that selected *SLC11A1* polymorphisms did not affect susceptibility to CL in the sample population. This work can be further extended to incorporate other polymorphisms of *SLC11A1* and as well as other immune regulatory genes and observe their combined result. The analysis of host genetic factors will help understand better the molecular mechanisms involved in mediating control and elimination of disease parasites which may explain the difference in susceptibility among different populations.

## Additional file

Additional file 1: Table S1. Genetic variants identified within *SLC11A1* gene through exon specific amplification and restriction fragment length polymorphism (PCR-RFLP) analysis. (DOCX 16 kb)

## Abbreviations

ACD: Acid citrate dextrose
CL: Cutaneous leishmaniasis
dNTPs: Deoxynucleotide triphosphates
HIV: Human immunodeficiency virus
LD: Linkage disequilibrium
MAF: Minor allele frequencies
MEGA: Molecular evolutionary genetics analysis
OR: Odds ratio
PCR: Polymerase chain reaction
PCR-RFLP: Polymerase chain reaction-restriction fragment length polymorphism
RFLP: Restriction fragment length polymorphism
SLC11A1: Solute carrier family 11 member a1
SNP: Single nucleotide polymorphism
UTR: Untranslated region
VL: Visceral leishmaniasis
