# Supplementary material for: SLC11A1 polymorphisms and host susceptibility to cutaneous leishmaniasis in Pakistan
Source: Parasit Vectors. 2017 Jan 7;10:12. doi: 10.1186/s13071-016-1934-2 (PMC5219684; doi:10.1186/s13071-016-1934-2)
Supplement: Additional file 1: Table S1. — Genetic variants identified within SLC11A1 gene through exon specific amplification and restriction fragment length polymorphism (PCR-RFLP) analysis. (DOCX 16 kb) [file 13071_2016_1934_MOESM1_ESM.docx]

**Additional file 1. Table S1.** Genetic variants identified within *SLC11A1* gene through exon specific amplification and restriction fragment length polymorphism (PCR-RFLP) analysis.

| Region | Primers 5`-3` (^a^F and ^b^R) | Polymorphism | Allele | Restriction enzyme | Digestion products [bp] |
| --- | --- | --- | --- | --- | --- |
| Exon 3 | **F**: GGCTGATGAGCCTGTTGG  **R**: CAGGGGGCCTTCAACACTTAG | 274C/T | C/T | *Mnl*1 | C : 95, 65, 51, 38, 33, 12, 4  T : 160, 51, 38, 33, 12, 4 |
| Intron 5 | **F:** AGGAGGCCAGATTCCTGTCT  **R:** TTCGATGTCAGAGCCCTTCT | 577-18G/A | A/G | *Msp*I | A: 240  G: 166, 74 |
| Exon 9 | **F:** GTGAGGGTGGGGGACACT  **R:** GGTCCTCGGCAAGGCTTA | A318V | T/C | *Fnu4H*I | T: 297  C: 224, 73 |
|  |  | 825A/G | A/G | *BsrB*I | A: 275  G: 175, 101 |
| Exon 15 | **F:** GCATCTCCCCAATTCATGGT  **R:** AACTGTCCCACTCTATCCTG | D543N | A/G | *Eco*471 | G: 201, 39  A: 126, 79, 39 |
| 3′UTR | Same as Exon 15 | 1729+55del4 | +TGTG  -TGTG | *Fok*I | +TGTG: 211, 33  -TGTG: 240 |

^a^ = forward primer

^b^ = reverse primer

Primers for exon 15 and 3’UTR region were described by Liu et al [17].
